# Letter to the editor regarding: ‘bidirectional association of daily steps with sarcopenia: a longitudinal study’

**DOI:** 10.1080/07853890.2025.2604394

**Published:** 2025-12-22

**Authors:** Chenxi Luo, Jing Luo, Shuang Wu

**Affiliations:** a The First School of Clinical Medicine, Zhejiang Chinese Medical University, Hangzhou, Zhejiang, China; b The Third Affiliated Hospital of Zhejiang, Chinese Medical University (Zhongshan Hospital of Zhejiang Province), Hangzhou, Zhejiang, China; c The Third School of Clinical Medicine (School of Rehabilitation Medicine), Zhejiang Chinese Medical University, Hangzhou, Zhejiang, China

Dear Editor

We read with interest the article ‘Bidirectional association of daily steps with sarcopenia: a longitudinal study’ published in your journal [1]. The study offers valuable insights into step-based interventions for sarcopenia and its potential reversibility, with implications for both clinical practice and research. However, several methodological aspects warrant further discussion.

First, step counts were collected *via* WeChat on smartphones, which may be less convenient for sarcopenic patients to carry consistently. We suggest the ActiGraph-GT3X accelerometer as a more reliable alternative. Prior research indicates that smartphone-based step counts tend to exceed those from ActiGraph devices [2]. Demographic variables such as age, gender, and education may also affect the reliability of smartphone data. The ActiGraph-GT3X is a validated reference tool for real-life physical activity assessment and is more suitable for older adults or those less familiar with technology [3]. Its use could reduce bias in studies examining step counts and sarcopenia.

Second, the daily step count was monitored over only seven days. This short duration may not sufficiently reflect long-term walking patterns. Physical activity, as indicated by step counts, and the development of sarcopenia both change over time. A longer monitoring period would therefore help clarify their bidirectional relationship. We recommend extending the step count recording phase and carefully planning the number and spacing of assessments. This approach would yield more stable data and improve insight into the association between daily steps and sarcopenia.

Third, dichotomizing sarcopenia status may obscure disease severity and limit clinical relevance. Furthermore, relying solely on grip strength to assess muscle strength may be confounded by comorbidities presenting with similar symptoms. We recommend that future studies adopt a graded classification of sarcopenia severity and incorporating additional measures such as chair stand tests, short-distance walks, timed up-and-go, and 400-meter walk tests to improve diagnostic comprehensiveness [4].

Fourth, although a logistic regression model was used to examine the step–sarcopenia association, it remains unclear whether univariate analysis or chi-square tests were performed for variable selection prior to multivariate modeling. In observational data analysis, univariate screening and chi-square tests help prevent overfitting, maintain model goodness-of-fit, and enhance robustness. We recommend that future studies explicitly describe and discuss such preliminary analyses.

In summary, Wang et al.’s study [1] provides a valuable foundation for future research. We recommend using ActiGraph accelerometers, extending step monitoring periods, refining sarcopenia assessments with multiple physical tests, and performing preliminary univariate analyses. These improvements could help clarify causal mechanisms and support public health strategies for sarcopenia prevention.

## Data Availability

Data sharing does not apply to this article, as no data were created or analysed in this study.
